# Effective capture of circulating tumor cells from an S180-bearing mouse model using electrically charged magnetic nanoparticles

**DOI:** 10.1186/s12951-019-0491-1

**Published:** 2019-05-04

**Authors:** Zhiming Li, Jun Ruan, Xuan Zhuang

**Affiliations:** 1 Institue of Reproductive Health, Tongji Medical College, Huazhong University of Science and Technology, Wuhan, 430030 Hubei China; 2 Department of Urology, The First Affiliated Hospital of Xiamen University, Xiamen, 361003 Fujian China; 3 College of Life Sciences, Central China Normal University, Wuhan, 430079 Hubei China; 4 Department of Clinical Medicine, Fujian Medical University, Fuzhou, 350005 Fujian China

**Keywords:** Circulating tumor cells, Cell surface charge, S180-bearing mouse, Nanoparticles

## Abstract

**Background:**

Technology enabling the separation of rare circulating tumor cells (CTCs) provides the potential to enhance our knowledge of cancer metastasis and improve the care of cancer patients. Modern detection approaches commonly depend on tumor antigens or the physical size of CTCs. However, few studies report the detection of CTCs by the electrical differences between cancer cells and normal cells.

**Results:**

In this study, we report a procedure for capturing CTCs from blood samples using electrically charged superparamagnetic nanoparticles (NPs). We found that only positively charged NPs attached to cancer cells, while negatively charged NPs did not. The capture method with positively charged NPs offered a sensitivity of down to 4 CTCs in 1 mL blood samples and achieved a superior capture yield (> 70%) for a high number of CTCs in blood samples (10^3^–10^6^ cells/mL). Following an in vitro evaluation, S180-bearing mice were employed as an in vivo model to assess the specificity and sensitivity of the capture procedure. The number of CTCs in blood from tumor-bearing mice was significantly higher than that in blood from healthy controls (on average, 75.8 ± 16.4 vs. zero CTCs/100 μL of blood, p < 0.0001), suggesting the high sensitivity and specificity of our method.

**Conclusions:**

Positively charged NPs combined with an in vivo tumor model demonstrated that CTCs can be distinguished and isolated from other blood cells based on their electrical properties.

**Electronic supplementary material:**

The online version of this article (10.1186/s12951-019-0491-1) contains supplementary material, which is available to authorized users.

## Background

Circulating tumor cells (CTCs) are cells that shed from primary tumors into the blood and are carried around the body by the circulation. It has been accepted that CTCs constitute seeds for the tumor metastasis, which is responsible for the majority of cancer-related death. “Liquid biopsy” via detection of CTCs from peripheral blood is a promising alternative method to determine tumor progression and metastasis. However, CTCs are extremely rare, with roughly one CTC per billion blood cells, in cancer patients [1]. The only FDA-approved CTC detection platform, CellSearch, is dependent on epithelial cell adhesion molecule (EpCAM). It has been shown that cancer cells express a variety of molecular proteins in a dynamic fashion, which complicates the separation of CTCs. This conclusion is supported by clinical data showing that CellSearch exhibited low detection efficiency in non-small cell lung cancer [2]. Cell filtration and centrifugal force devices are alternate platforms to isolate CTCs from blood cells and are based on the assumption that tumor cells are larger and less deformable than normal haematological cells. However, CTCs of various sizes have been identified by many CTC detection methods, including CellSearch. Thus, more sophisticated label-free microfluidics approaches involving dielectrophoresis (DEP) or magnetophoresis (MAP) have recently been utilized [3].

Approximately 100 years ago, Cure et al. found that cancer cells have extraordinarily high concentrations of negatively charged glycoproteins on their exterior surface, which act as an electrical shield [4, 5]. Sialic acid is considered to be one of the primary molecules responsible for conferring a negative charge to glycoproteins. Since human chorionic gonadotropin (hCG) contains large amounts of sialic acid, this results in cancer cells having a stronger negative cell surface charge than normal cells [6]. Moreover, cancer cells have highly altered energy metabolism, including increased reliance on glycolysis and a shift to the use of glutamine in the tricarboxylic acid cycle (TCA) cycle. It is estimated that cancer cells produce up to 40 times more lactic acid than normal cells [7]. Lactic acid is not electrochemically active, but nicotinamide adenine dinucleotide (NADH), the enzyme cofactor driving the reduction of pyruvate to lactate via lactic acid dehydrogenase, is oxidized to NAD+, which is electrochemically active at sufficiently negative potentials [8]. Jeffrey recently observed that acute lymphoblastic lymphoma T-cells (T-ALL cells) exhibit a faradaic electrochemical response that is two orders of magnitude greater than that of normal cells [9].

In this paper, we designed fluorescent superparamagnetic Fe_3_O_4_ composite nanoparticles with electrically charged surfaces to detect cancer cells without relying on cancer cell biomarkers. The negatively charged nanoparticles did not bind to cancer cells because of the electrostatic repulsion between them. However, the positively charged nanoparticles effectively captured cancer cells from blood samples in vitro and S180-bearing mice in vivo. This study provides evidence that the electrically properties of cancer cells can be used as a unique feature to capture CTCs from the circulating blood.

## Results

### Preparation and characterization of electrical NPs

**Fig. 1:**
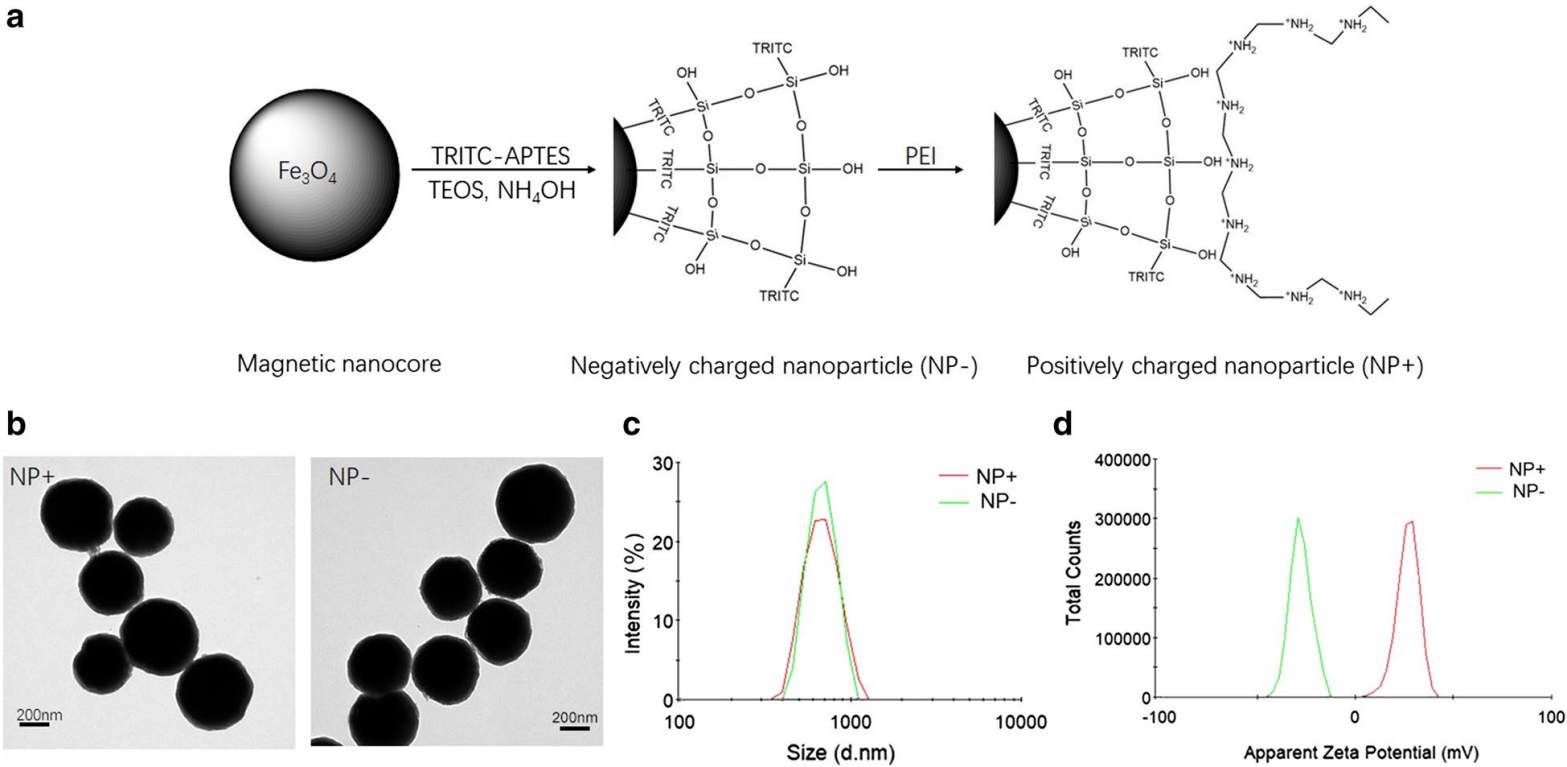
Design and characterization of the nanoparticles. **a** Schematic diagram showing the design of surface-charged, fluorescent, superparamagnetic composite nanoparticles (NPs). **b** Transmission electronic microscopy image of positively charged nanoparticles (NP+) and negatively charged nanoparticles (NP−). **c** Dynamic light scattering analysis showing size and distribution of NPs. **d** Zeta potential distributions of NPs

A schematic diagram for preparation of the surface-charged magnetic composite nanoparticles is displayed in Fig. 1a. As shown in Fig. 1a, the Fe_3_O_4_ microspheres are conjugated with (3-aminopropyl) triethoxysilane (APTES) to form a thin SiO_2_ shell layer on the surface of microspheres upon reaction with tetraethyl orthosilicate (TEOS) and ammonium hydroxide (NH_4_OH). To directly visualize and quantify captured cells, an APTES-tetramethylrhodamine (TRITC) complex is initially reacted, followed by grafting onto the surface of the Fe_3_O_4_@silica composites through a classical sol–gel reaction. Abundant SiOH groups govern the overall surface of this product: negatively-charged nanoparticles (NP−), exhibiting a strong negative surface charge. For positively-charged nanoparticles (NP+), polyethyleneimine (PEI) molecules are used to cover and modify the surface of NP−. The modified product shows a strong positive surface charge due to the abundant presence of amine groups.

**Fig. 2:**
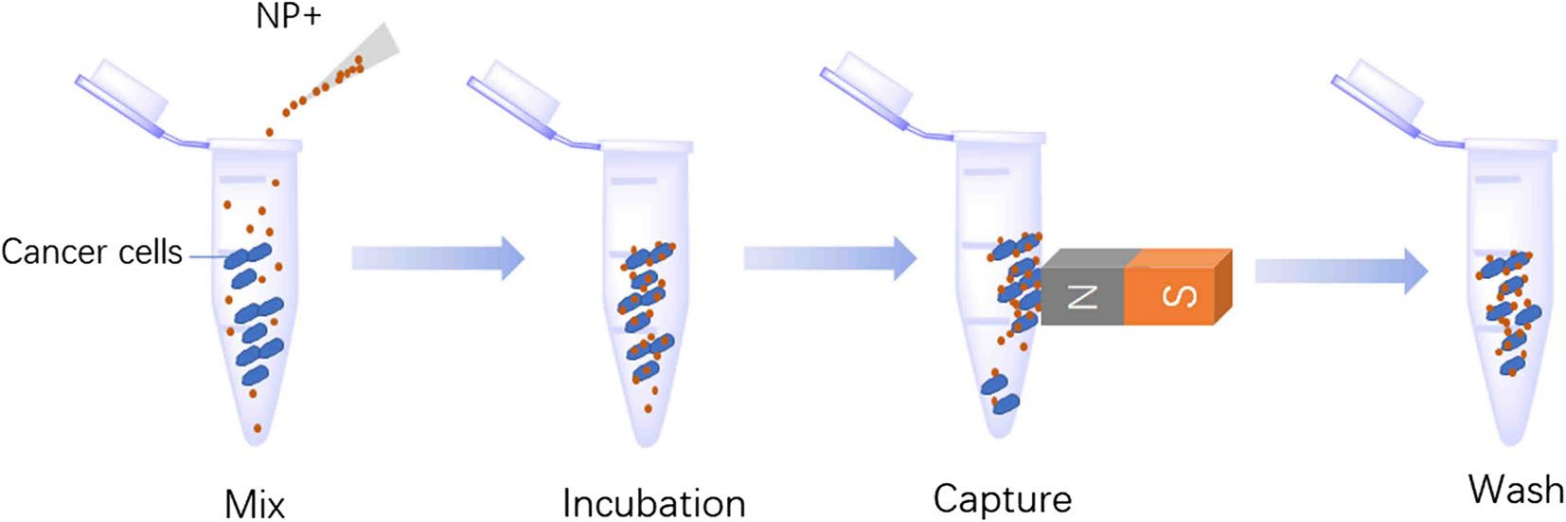
Illustration of the procedures for cancer cell capture. Cancer cells in suspension were mixed with NP+ and NP−, followed by 10 min of incubation. The “magnetized” cancer cells were pulled onto the wall of the vial using a magnet. After removal of the remaining solution, the cells were captured with NPs, washed and counted

As shown in Fig. 1b, the transmission electron microscopy (TEM) demonstrated that the magnetic composite nanoparticles had a diameter of 450 nm and exhibited a uniform SiO_2_ coating (size of 60 nm). Dynamic light scattering (DLS) analysis of the particles showed a narrow size distribution with an increased average diameter of 620 nm after surface functionalization (Fig. 2b). Figure 1c shows the zeta potential distribution of the negative and positive nanoparticles. In deionized water (pH 7.0), the zeta potentials of the NP− and NP+ are − 26.6 mV and + 28.1 mV, respectively. These results indicated that the surface-charged nanoparticles were well dispersed in aqueous solution under neutral conditions and thus could be applied for cell capture.

### Capture of cancer cells independent of surface protein expression

Figure 2 shows the general experimental procedure for cell capture. NPs were mixed with a solution of cancer cells and incubated at room temperature for 10 min. Subsequently, we used a permanent magnet to capture the “magnetized” cells (magnetic nanoparticles bound to the cell surface) onto the wall of the tube. After removal of the remaining solution and washing of the aggregates with PBS (with a magnet outside the tube), we transferred the aggregates to a haemocytometer for quantification and to a slide for validation.

**Fig. 3:**
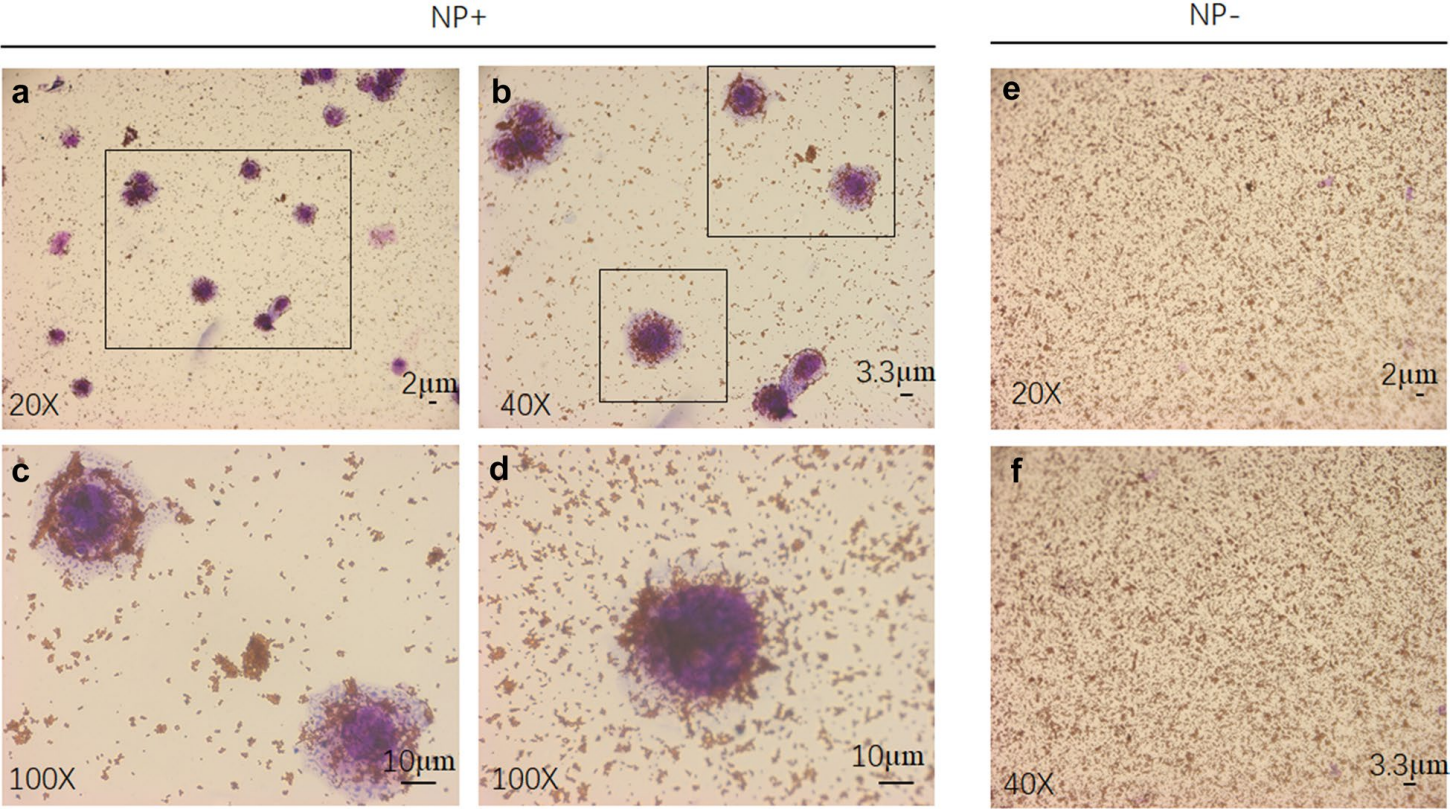
Comparison of MDA-MB-231/GFP cell binding capacity of NP+ and NP− in PBS. Phase-contrast images (**a**, **b**, **c**, **d**) of NP+ bound to MDA-MB-231/GFP cells (left). The right images (**e**, **f**) only show NP−, revealing a lack of binding capacity to MDA-MB-231/GFP cells

**Fig. 4:**
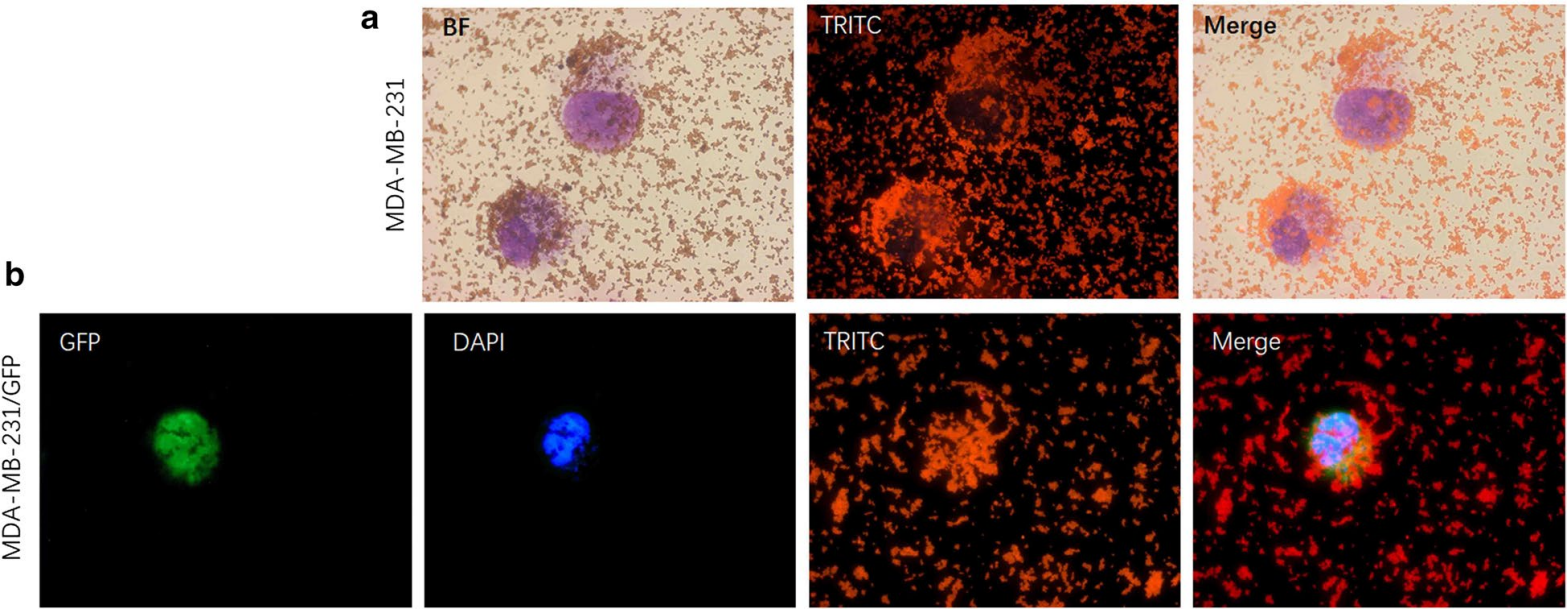
Fluorescence images of NP+ bound to MDA-MB-231/GFP cells in PBS. **a** Phase-contrast and fluorescence images of MDA-MB-231/GFP cells bound with NP+. **b** A representative fluorescence image of a captured cell. DAPI was used to stain cell nuclei. TRITC was used to label the NPs. *BF* Bright field

We found that NP+ indeed interacted with cancer cells in PBS solution. In the spiked cancer cell capture experiments, MDA-MB-231/green fluorescent protein (GFP) cells were used as targets. The cultured cells were detached with trypsin, washed with PBS, and resuspended in PBS. Figure 3a shows an optical image of cancer cells captured by NP+. Under high magnification of this image, we found that a large number of NP+ were attached on the surface of MDA-MB-231/GFP cells, as shown in Fig. 3b–d. When cancer cells were captured with NP− under the same experimental conditions, we did not see any cancer cells in the image, only a great number of nanoparticles, as shown in Fig. 3e, f. These results demonstrated that NP+ and NP− had completely different patterns of interaction with cancer cells, suggesting a negatively charged surface of the cancer cells. A bright field image of a typical captured cell shown in Fig. 4a and illustrates a near-spherical shape of the cells with labelled particles on the surface edge. The fluorescence field illustrated that the spherical shape around the cells was composed of NP+, shown in red (from TRITC). To further characterize the interaction of NP+ and cells, a fluorescence analysis was used for direct imaging of the NPs bound on the cells. Fluorescence images of the cells (Fig. 4b) revealed that captured MDA-MB-231/GFP cells were positive for 4′,6-diamidino-2-phenylindole (DAPI, blue), GFP (green) and TRITC (red), suggesting that NP+ bind to the cancer cells via a specific interaction.

**Fig. 5:**
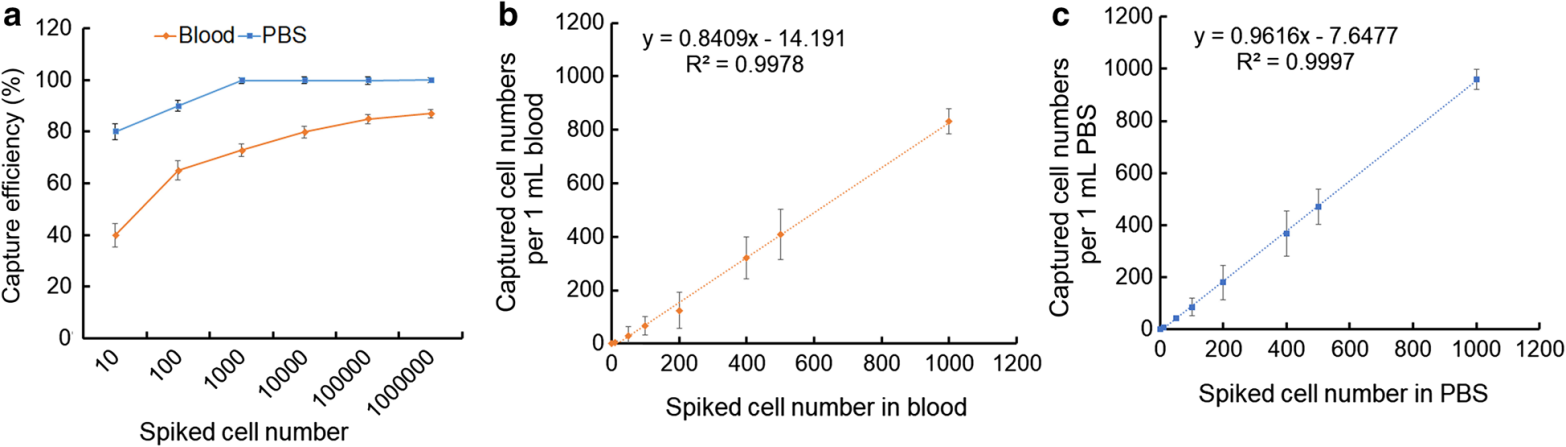
Detection analysis of CTCs from in vitro spiked samples. **a** The capture efficiency of MDA-MB-231/GFP cells using NP+ in PBS and whole blood spiked with different numbers of cells (concentrations ranging from 10 to 10^6^ cells/mL). Regression analysis of capture efficiency in whole blood (**b**) and PBS (**c**)

We next compared the capture rate between 1 mL PBS and 1 mL blood spiked with the same number of MDA-MB-231/GFP cells using NP+. Figure 5a shows that over 80% and 99% of CTCs can be remarkably isolated from PBS spiked with a low number of cells (10–10^2^) and a high number of cells (10^3^–10^6^), respectively. For the blood sample, the capture ratios were > 40% for 10–10^2^ cancer cells and > 70% for 10^3^–10^6^ cancer cells. Strong linear correlations between the number of cancer cells captured vs. the number of cancer cells initially loaded (n = 10–10^6^) were observed for both blood and PBS samples (Fig. 5b, c). Taken together, our results showed that NP+ can achieve efficient capture of CTCs, which is independent of protein expression on the cell surface. Based on the linear correlation, this method can be used to quantify CTC numbers in mouse blood for CTC numbers higher than 4 cells per a 1 mL blood sample.

To investigate the optimized charge that allows NP+ to separate cancer cells from healthy cells, capture efficiencies of NP+ with different charges were analysed. We found that nearly all S180 and MDA-MB-231 cancer cells were captured at a zeta potential of + 25 mV, while normal white blood cells (WBCs) were not (Additional file 1: Figure S1). Additionally, we observed that a small number of WBCs were simultaneously enriched with the cancer cells. Given that a phagocytosis effect could be caused by phagocytes, we isolated human neutrophils (the most abundant type of phagocyte in the bloodstream) from whole blood using the density gradient separation method [9]. Intracellular accumulation of nanoparticles obviously presented when a large number of neutrophils (10^5^) were incubated with NP+ (Additional file 1: Figure S2).

### Capture of CTCs from the S180-bearing mouse model

**Fig. 6:**
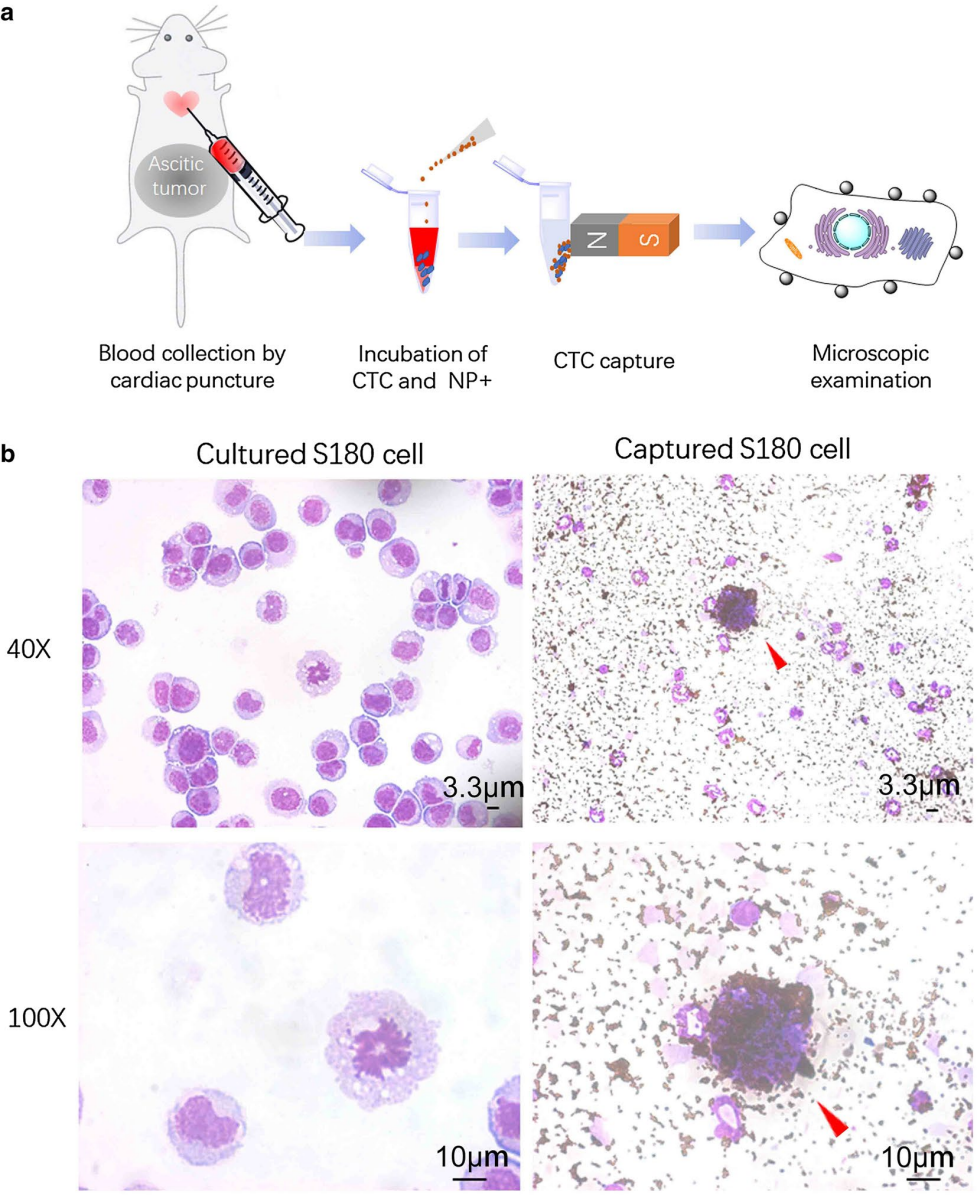
CTC detection in blood from S180-bearing mice using NP+. **a** An illustration of the capture assay using NP+ in the animal model. **b** Optical images of S180 cells under normal culture conditions. Representative staining images showing CTCs captured from mice with S180 ascitic tumors

We tested the CTC capture procedures using NP+ in an S180-bearing mouse model of sarcoma. To generate ascitic tumors, 2 × 10^6^ S180 cells were i.p. injected into C57BL/6 mice. When ascitic tumor growth was observed within 2–3 weeks, the mice were euthanized according to the standard IACUC procedures. Nearly 200–500 µL blood was collected from each mouse via cardiac puncture of the left ventricle. We mixed 30 µg NP+ with 100 µL whole blood and then detected CTCs according to the method described above. Figure 6a shows the general experimental procedure. The cells were captured by the NP+, washed with PBS, and stained with HEMA-3. Figure 6b shows the typical shape of S180 cells under normal culture conditions and an aliquot of captured cells in the S180-bearing mouse blood sample. The red arrow marks the unique cells that have a high level of NP+ bound to the cell surface. The general diameter of S180 cells is approximately 50 µm, which is much larger than that of white blood cells (12–20 µm), such as, granulocytes, lymphocytes, or monocytes, as shown in Fig. 6b. In addition to the cell surface being densely decorated by particles, physical size was utilized to discriminate CTCs from nonspecific cells in tumor mouse blood. Moreover, we established a stable cell line that constitutively express GFP-tags to detect and track CTCs from S180-bearing mice. The results showed that the captured CTCs were double-positive (TRITC/GFP) and polyploidy tumor cells, which are indeed tumor cells excreted from the primary tumors (Additional file 1: Figure S3).

**Fig. 7:**
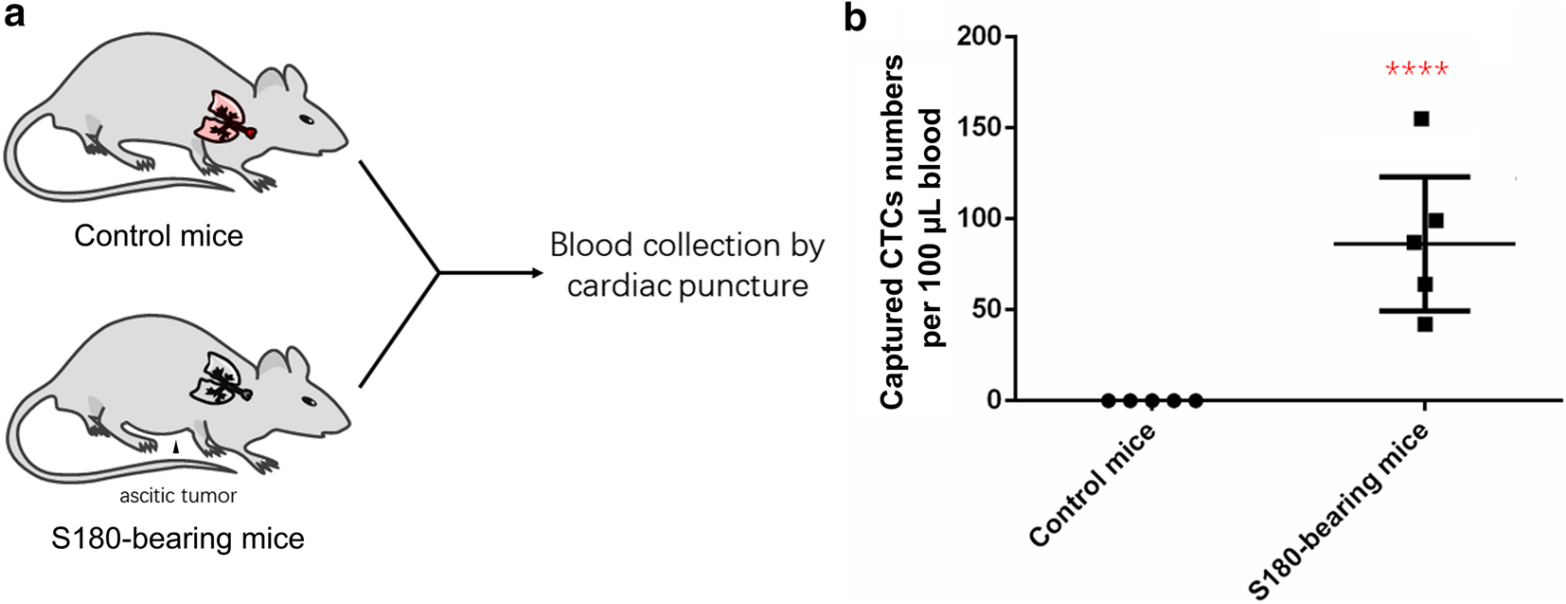
Comparison of captured CTCs from healthy and tumor-bearing mice using NP+. **a** An illustration of the experiment using wild-type mice and S180-bearing mice. **b** Comparison of the captured cell numbers per 100 µL blood between the two groups. A significantly higher number of CTCs was captured from the S180-bearing mice than the control mice (****p < 0.0001)

To evaluate the applicability of our CTC detection platform as a diagnostic tool, five healthy mice and five S180-bearing mice were used (Fig. 7a). After 2 weeks of ascitic tumor growth, the mice were sacrificed, and whole blood was collected via cardiac puncture before being processed with the CTC capture system using NP+. The number of captured CTCs per 100 μL of blood from both healthy and S180-bearing mice with sarcoma tumors is plotted in Fig. 7b. Significantly more CTCs were captured from the S180-bearing mice with sarcoma (75.8 ± 16.4 CTCs per 100 μL, n = 5) than from the wild-type controls (no CTCs observed per 100 μL, n = 5, p < 0.0001), demonstrating the applicability of this method to detect in vivo CTCs. The successful validation of our CTC detection system using S180-bearing mice confirmed that it can overcome the limitations of in vitro evaluations, the heterogeneity of surface marker expression on CTCs and the high number and variety of haematological cells in the blood. Compared with CellSearch, our method detected significantly higher positive rates in samples from 15 metastatic breast cancer patients (Additional file 1: Figure S4).

## Discussion

Due to the specific metabolic pattern of cancer cells that exhibit the Warburg effect, we posit that using a CTC capture system that is electrically focused and charge-based will have greater success in detecting CTCs with different molecular signatures. This fact was supported by our results: positively charged NPs were capable of capturing heterogeneous CTC populations independent of their surface marker expression. Specifically, using the CTC capture assay with NP+, we were able to achieve capture yields of > 70% and > 90% of MDA-MB-231 breast cancer cells (10^3^–10^6^) spiked in 1 mL whole blood samples and PBS, respectively. For very low numbers of spiked cancer cells (10–10^2^ cells), our technique was capable of detecting the presence of at least 4 and 8 cancer cells in whole blood samples and PBS, respectively, suggesting that the electrical particles can be effectively applied to capture cancer cells for in vivo studies. Data pooled from the S180-bearing mouse models showed that the number of CTCs captured by NP+ ranged from 35 to 167 cells per 100 μL of blood at the 2-week point in tumor progression. All these results demonstrated that our method has a higher capacity to capture tumor cells and shows better results than currently reported dendrimer-immobilized biomarkers or microfluidic chip techniques for separation of rare cells [10, 11].

Sarcomas are cancers of the bone and connective tissues that arise from transformed cells of mesenchymal origin. Bone sarcomas and soft tissue sarcomas are two main sarcoma subtypes. The S180 cell line was initially isolated from a soft tissue tumor in a Swiss mouse. When implanted into the peritoneal cavity, S180 cells gradually plug peritoneal lymphatic drainage, inducing accumulation of ascites fluid within 2–3 weeks [12–14]. S180 cells also exhibit metastasis to major organs near the peritoneal cavity. Sarcoma metastasis is correlated with the number of CTCs in peripheral blood, which is supported by our observation of the substantially higher number of CTCs in the S180-bearing mice than in the wild-type mice. Thus, S180-bearing mice with a higher number of CTCs in peripheral blood provides a good model to evaluate a CTC capture assay. The in vivo results illustrate the promise of our CTC detection platform for eventual use in human tumor detection. Moreover, we can also apply our CTC detection platform to monitor the therapeutic approach for treatment of cancer in animal models or clinical studies.

Previously, it has been noted that cancer cell membranes have a higher number of negatively charged sialic acids, which ultimately determines a cell’s zeta potential [15]. Recent studies confirmed that the fate of cancer cells can be controlled by altering the electrical charge of the cell membrane [4, 16]. In our study, NP+ were found to be attracted to both MDA-MB-231 and S180 cancer cells, but NP− captured neither of these cell types, which further confirms that NP+ bind to cancer cells via a specific interaction. In 1995, via electrorotation, Becker et al. investigated the electrical properties of the MDA-MB-231 breast cancer cell line in contrast with erythrocytes and T lymphocytes [17]. Again, we can see that based on the electrical properties of cancer cells, CTCs can be separated from other blood cells. Our magnetic NPs can be moved inside a microfluidic channel by applying an inhomogeneous magnetic field, which makes the magnetophoretic sorting process highly sensitive.

## Conclusions

In summary, we developed a new strategy for cell surface-charge-based CTC capture by exploiting the elevated negative charge of cancer cells that is independent of CTC marker expression or epithelial–mesenchymal state, both of which change throughout tumor development and metastasis. We demonstrated that our CTC capture method is effective and applicable to detect CTCs in vitro and in vivo, thus offering a promising method to monitor cancer progression and responsiveness to therapeutic intervention.

## Materials and methods

### Nanomaterials

Iron (III) chloride hydrate (FeCl_3_·6H_2_O), NH_4_OH (28 wt%), hydrochloric acid (37 wt% aqueous solution), ethylene glycol and sodium acetate were purchased from Shanghai (China) Reagent Company. TEOS, APTES and TRITC were purchased from Sigma-Aldrich (USA). PEI (99%, Mw = 10,000) was purchased from Alfa Aesar. All the solutions were prepared using Milli-Q deionized water (18.2 MΩ cm at 25 °C resistivity).

### NP synthesis

Magnetic microsphere cores were produced via a solvothermal reaction. Briefly, 0.081 g of FeCl_3_·6H_2_O was dissolved in 30 mL of ethylene glycol under magnetic stirring. Then, 0.3 g of polyacrylic acid (PAA) and 1.8 g urea were added to this solution. After being stirred for 30 min, the solution was heated at 200 °C for 12 h using a Teflon-lined stainless-steel autoclave. When cooled to room temperature, a black product, namely, Fe_3_O_4_ microspheres, was collected with a magnet. Following washes with ethanol and deionized water three times each, the Fe_3_O_4_ microspheres were treated with 0.15 M HCl under sonication for 15 min and then coated with silica via hydrolysis and condensation of TEOS.

To prepare the negatively charged fluorescent magnetic nanoparticles, an APTES-TRITC complex was first reacted under dark conditions overnight in ethanol. The complex was then grafted to the Fe_3_O_4_ microspheres through reaction between APTES and hydroxyl groups on the Fe_3_O_4_@SiO_2_ microspheres. Subsequently, 30 µL of TEOS was added and reacted for 24 h in the dark. Following washes with ethanol and deionized water three times each, NP− were produced. Through modification of NP− with the polycation polymer PEI, positively charged magnetic nanoparticles (NP+) were obtained.

### NP characterization

TEM studies were performed using a TECNAI F-30 high resolution transmission electron microscope operating at 300 kV. Surface morphology and structure of the particles were examined using a field emission scanning electron microscope (FE-SEM, S-4300, HITACHI, Japan). The particle size and zeta potential of NPs were determined with a Malvern Zeta Sizer Nano series (Westborough, MA). Fluorescence was examined with a Carl Zeiss LSM5 EXITER laser scanning confocal microscope (Zeiss, Jena, Germany).

### Patients

We obtained peripheral blood samples from 15 patients with metastatic breast cancer who presented themselves at the clinic untreated. All patients gave informed consent for the use of their blood specimen, and examination of blood samples was carried out after approval from the institutional review board. The median age of the control population was 52 (range 34–75) years. For each patient, 10 mL of blood was collected in EDTA tubes for CTC enumeration.

### Cell culture

We previously transduced human breast cancer MDA-MB-231 cells with a lentiviral construct containing GFP as a reporter. Using fluorescence activated cell sorting, we established a stable cell line (denoted MDA-MB-231/GFP) that expresses high levels of GFP for at least 12 passages in culture. The human breast cancer-derived cell line MDA-MB-231/GFP and murine sarcoma 180 (S180) cell line were cultured in Dulbecco’s modified Eagle’s medium High-Glucose (DMEM, Gibco) supplemented with 10% foetal bovine serum (FBS, Gibco).

### Murine metastasis model

Ascites was induced in C57BL/6 mice via intraperitoneal (ip) injection of a 2 × 10^6^ S180 cell suspension in 1 mL PBS. The mice were inspected and weighed daily for assessment of ascites development. All of the animal research procedures were approved by the Institutional Animal Care and Use Committee.

### Blood collection

Mouse blood samples (200–500 µL) were obtained via cardiac puncture and collected into K2-EDTA-coated tubes. To perform cardiac puncture, mice were deeply anaesthetized under isoflurane, and a 21-gauge needle coated with heparin was inserted into the heart. Mice were euthanized immediately following the cardiac puncture.

### CTC capture from in vitro spiked samples

The total cancer cell number was first quantified with an automated cell counter (Invitrogen Countess, US) and then confirmed with a haemocytometer. A 1:10 serial dilution of various numbers of MDA-MB-231/GFP cells was spiked into either a 1 mL volume of PBS or 1 mL of whole blood freshly harvested from healthy volunteers. Then, 30 µL of NPs (1 µg/µL) were added to the cell suspensions and incubated at room temperature for 10 min with gentle agitation. After incubation, the NP-bound cells were captured via a permanent magnet onto the wall of the vial, and free cells were removed with the remaining solution. The captured cells were released by removing the magnet and resuspended in PBS. We transferred captured cells to a haemocytometer for quantification. For microscopic analysis, an aliquot of cells was spread onto slides and stained with Haema-3 (Fisher Diagnostics).

### CTC capture from an in vivo S180-bearing mouse model

To detect the number of CTCs in mouse blood from wild-type C57BL/6 mice and S180-bearing mice with sarcoma tumors, five to six mice in each group were used. Blood for CTC detection was drawn via cardiac puncture. Capture of CTCs from mouse blood samples was performed using a procedure similar to the one employed for spiked blood samples. The researcher counting CTCs was blinded to the mouse group.

### Statistical analysis

The results are expressed as the mean ± standard deviation (SD) as indicated in the figure legends. Student’s two-sample, unpaired t-tests were calculated using GraphPad Prism software, with p-values < 0.05 considered statistically significant. Regression analysis and capture yield were analysed using Microsoft Excel software.

## Additional file

**Additional file 1.** Additional figures.

## Abbreviations

CTCs: circulating tumor cells
NPs: nanoparticles
EpCAM: epithelial cell adhesion molecule
DEP: dielectrophoresis
MAP: magnetophoresis
hCG: human chorionic gonadotropin
NADH: nicotinamide adenine dinucleotide
TCA: tricarboxylic acid cycle
T-ALL: acute lymphoblastic lymphoma T-cells
DLS: dynamic light scattering
DAPI: 4′,6-diamidino-2-phenylindole
TRITC: tetramethylrhodamine
PAA: polyacrylic acid
TEOS: tetraethyl orthosilicate
TEM: transmission electron microscopy
GFP: green fluorescent protein
NP+: positively-charged nanoparticles
NP−: negatively-charged nanoparticles
