# Supplementary material for: Effective capture of circulating tumor cells from an S180-bearing mouse model using electrically charged magnetic nanoparticles
Source: J Nanobiotechnology. 2019 May 4;17:59. doi: 10.1186/s12951-019-0491-1 (PMC6499951; doi:10.1186/s12951-019-0491-1)
Supplement: Supplementary file 1 — Additional file 1. Additional figures. [file 12951_2019_491_MOESM1_ESM.docx]

Additional Material for

**Effective capture of circulating tumor cells from an S180-bearing mouse model using electrically charged magnetic nanoparticles**

Zhiming Li, Jun Ruan, Xuan Zhuang


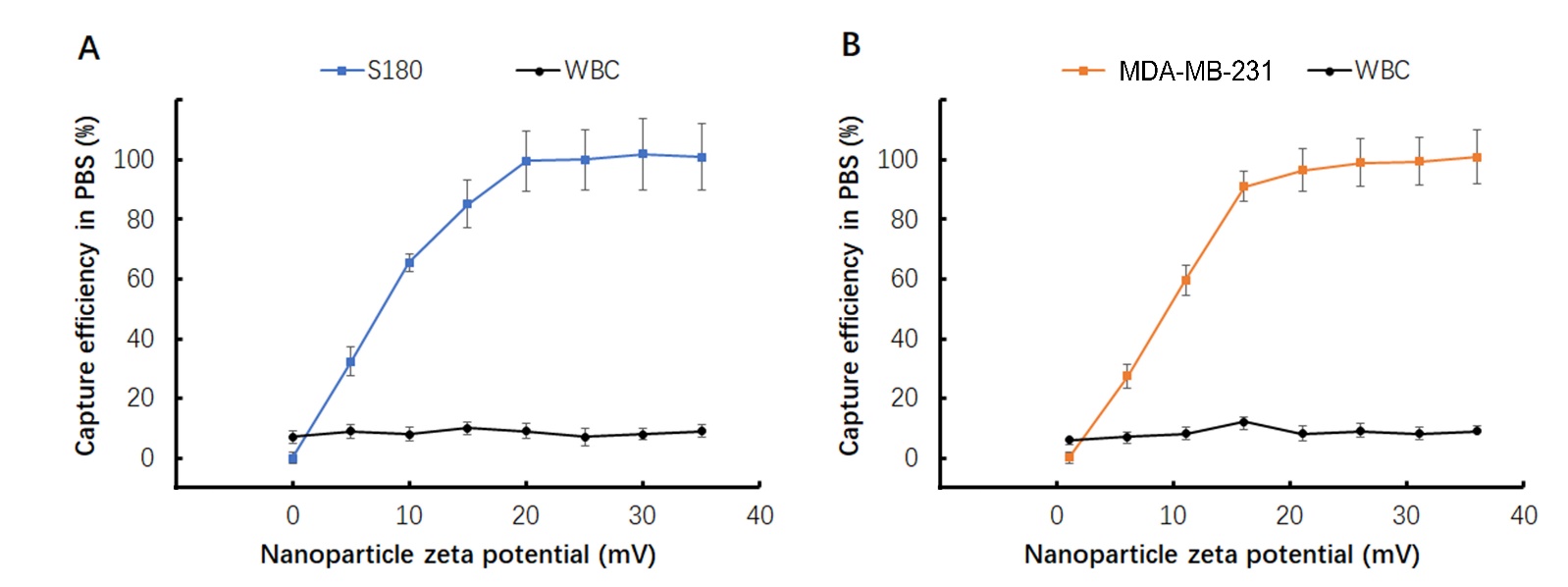


**Figure S1. Magnetic capture efficiencies of cancer cells using NP+ with different charges.**

S180 (A) and MCF7 (B) cancer cell capture efficiencies vs. normal white blood cells (WBC) when exposed to positive nanoparticles (NP+) with different charges.

To investigate the relationship between capture efficiency and the level of positive charge on the nanoparticles, we created eight types of nanoparticles with a gradient of positive surface charges (0, 5, 10, 15, 20, 25, 30, and 35 mV). The results show that the capture efficiency of cancer cells (10^3^) is indeed proportional to the level of positive surface charge. Over a zeta potential of +20 mV, nearly all S180 and MCF7 cancer cells were captured, while normal WBCs were not.


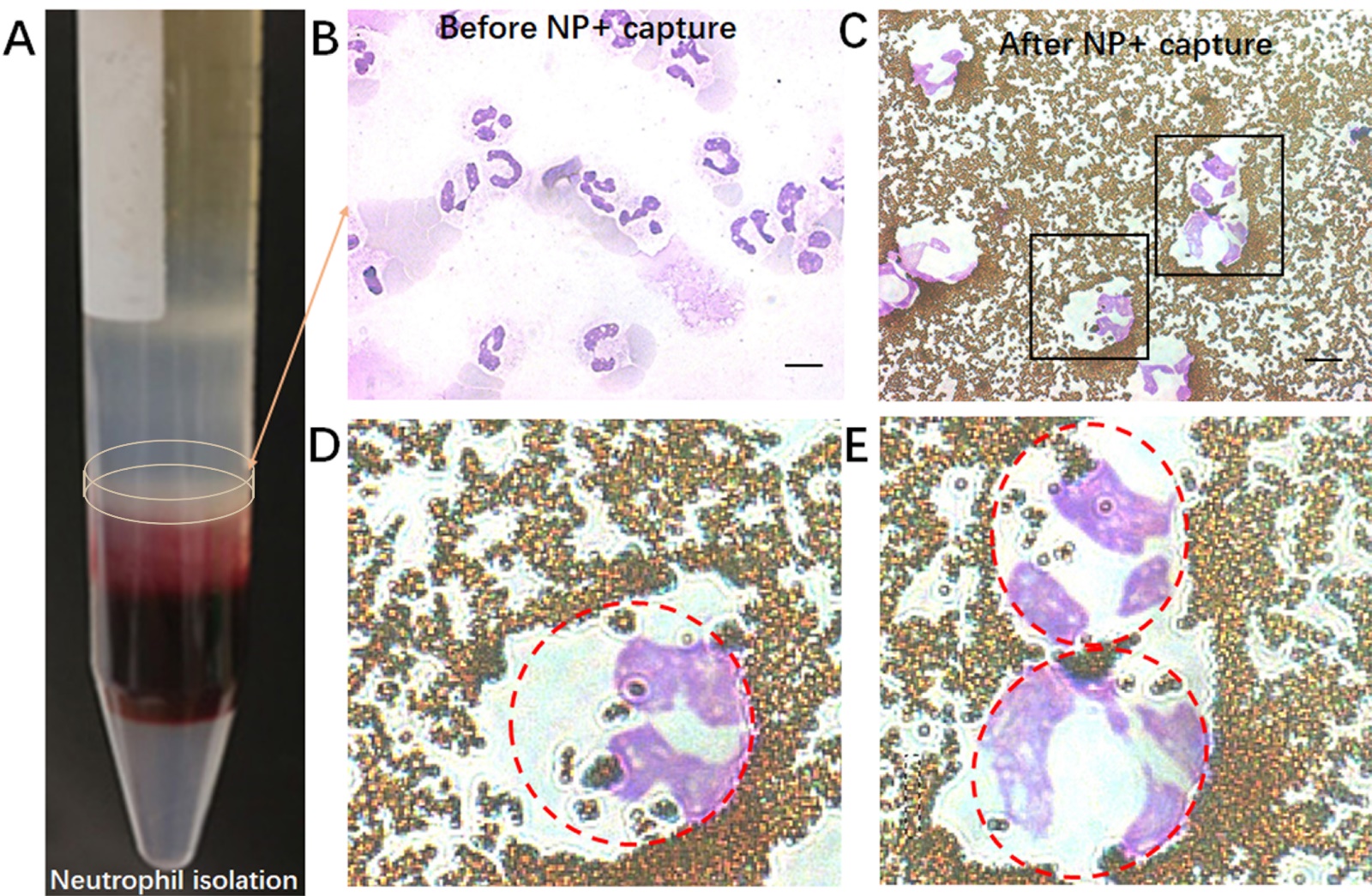


**Figure S2.** **Interaction of nanoparticles with neutrophils.**

(A) Photograph of plasma-Percoll® gradients following centrifugation. Neutrophils are located at the interface of the 42% and 51% layer. (B) Cytospins of neutrophils (10^5^ cells) from one healthy donor before and (C) after incubation with NP+. Scale bar = 10 µm. (D) and (E) High magnification images show intracellular accumulation of nanoparticles by neutrophils.

Neutrophils are normally found in the bloodstream and are the most abundant type of phagocyte, constituting 50-60% of the total circulating white blood cell population. To study the nanoparticle interactions with neutrophils, we isolated human neutrophils from whole blood using the density gradient separation method. Granulocytes (≥ 95% neutrophils) were located at the interface of the 42% and 51% layer (Figure S2A). Isolated cells were centrifuged onto a glass slide using a cytospin, air dried, fixed and stained with Hema-3. Most normal mature neutrophils exhibit ring-shaped nuclei with nuclear appendages and condensed chromatin (Figure S2B). Cells (10^5^) were mixed with NP+, washed, and stained with Hema-3. We observed intracellular accumulation of nanoparticles in neutrophils as denoted with dashed circles. (Figure S2C, 2D and 2E).


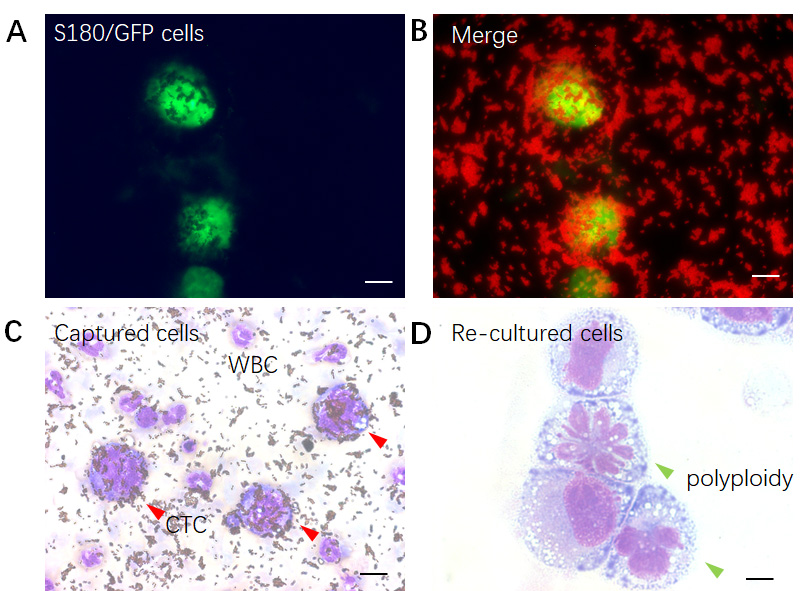


**Figure S3. The captured S180 CTCs are double-labelled and polyploid tumor cells.**

(A) Immunofluorescence image of S180/GFP cells. (B) A representative image of captured CTCs, which were double positive (TRITC/GFP). (C) Bright-field image of captured S180/GFP cells (red arrows) surrounded by NP+ after magnetic separation. (D) Two-day recultured cells exhibit nuclear polyploidy (green arrows). Scale bar = 10 µm.

No specific marker of S180 cells has been reported in the literature. Thus, we established a stable cell line that constitutively express GFP-tags to detect and track CTCs from S180 bearing mice (Figure S3A). Fluorescence microscopy images show cellular binding of NP+ (red) on the surface of S180/GFP cells (green). Optical microscopy images with Hema-3 staining show NP+ surrounding the edge of CTCs. Cell nuclei were analyzed using Hema-3 staining just after the cell had been released and recultured for 2 days. The nuclear polyploid structure of tumor cells was clearly observed.


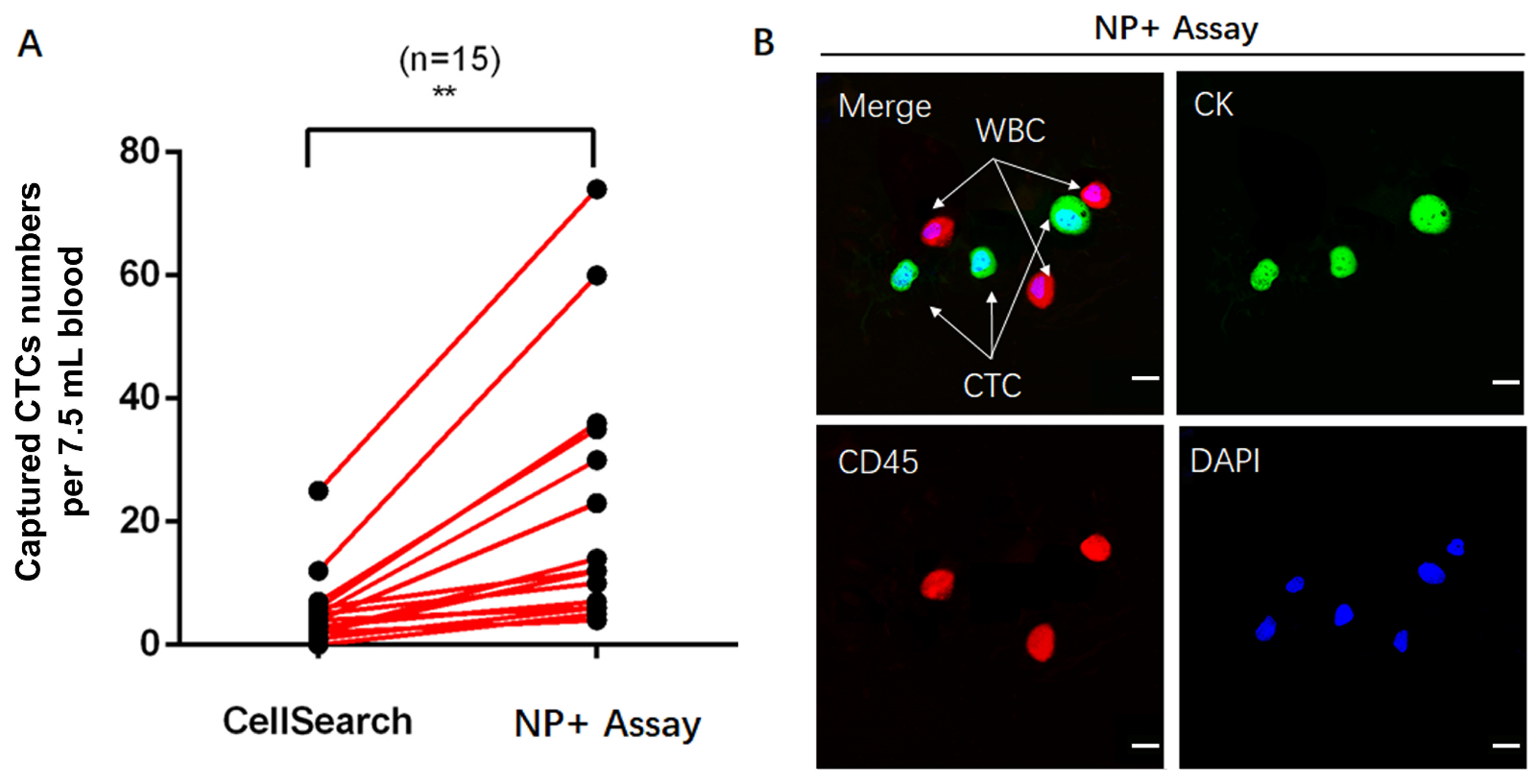


**Figure S4. Comparison of CTC detection using the CellSearch and NP+ methods.**

(A) Detection of CTCs in peripheral blood from metastatic breast cancer patients using either the CellSearch or NP+ assay. The results are normalized to a 7.5 mL scale for comparison. n = 15; **p < 0.001. (B) CTCs were captured by NP+ (without TRITC modification) and confirmed by the following criteria: CK+, CD45- and DAPI+. Scale bar = 10 µm.

To compare our CTC detection method with that of CellSearch (Janssen Diagnostics, Raritan, NJ), blood samples from 15 metastatic breast cancer patients were simultaneously analyzed using the two methods. The number of CTCs detected by the NP+ assay ranged from 5 to 74 (Figure S4A), while the number of CTCs detected by CellSearch ranged from 0 to 25. Higher CTC numbers were detected by the NP+ assay vs. CellSearch, and the CTC-positive rates were significantly higher in the former (p < 0.001). CTCs were enriched using the NP+ assay and stained with anti-CK and anti-CD45 monoclonal antibodies. CK+, CD45- and DAPI+ enriched cells were considered putative CTCs (Figure S4B). Additionally, a negligible number of CK-/CD45+ cells (10%) were detected in our enriched samples. They were considered to be WBCs and thus were not enumerated in the statistical analysis.
